# Prophylactic Antiseizure Medication in Patients with Lobar Intracerebral Hemorrhage

**DOI:** 10.1007/s12028-025-02423-w

**Published:** 2025-12-18

**Authors:** Margaret Banker, Juliana Silva Pinheiro do Nascimento, Harish Shownkeen, Tiffany R. Chang, Fernando Goldenberg, Brett Faine, David Cella, Stephan U. Schuele, Yuan Luo, Elizabeth Tipton, Andrew M. Naidech

**Affiliations:** 1 Department of Preventive Medicine, Feinberg School of Medicine, Northwestern University, Chicago, IL USA; 2 Institute for Public Health and Medicine, Feinberg School of Medicine, Northwestern University, Chicago, IL USA; 3 Northwestern Medicine Central Dupage Hospital, Winfield, IL USA; 4 Department of Neurosurgery, UTHealth Houston, Houston, TX USA; 5 Department of Neurology, University of Chicago, Chicago, IL USA; 6 Department of Emergency Medicine, University of Iowa, Iowa City, IA USA; 7 Department of Medical Social Sciences, Feinberg School of Medicine, Northwestern University, Chicago, IL USA; 8 Department of Neurology, Feinberg School of Medicine, Northwestern University, Chicago, IL USA; 9 Department of Statistics and Data Science, Northwestern University, Evanston, IL USA

**Keywords:** Acute intracerebral hemorrhage, Prophylactic antiseizure medication, Early seizures

## Abstract

**Background:**

Early seizures are a common complication after acute intracerebral hemorrhage (ICH). We tested the hypothesis of whether prophylactic antiseizure medication is associated with lower incidence of early seizures in patients with elevated risk of ICH.

**Methods:**

This study involved a retrospective analysis of a prospective observational cohort, including five academic medical centers with a focus on patients presenting spontaneous ICH on hospital admission in the years 2006 through 2023. We assessed the characteristics of acute ICH and the administration of antiseizure medication. In this observational cohort, the administration of antiseizure medication was at the discretion of the treating physician. We focused on the 300 patients with lobar hematoma location. Age, hematoma volume, and sex were included as covariates in an adjusted regression model to evaluate seizure occurrence. We prospectively recorded the use of antiseizure medications and identified the occurrence of early seizures. Additionally, we conducted an exploratory analysis defining patients who were at risk as those with lobar hematomas, age < 65 years, and hematoma volume ≥ 10 mL. Functional outcomes were assessed using modified Rankin Scale scores three months after event.

**Results:**

The median age was 72.0 (interquartile range 62.0–80.0) years, and 158 (53%) were female. An early seizure occurred in 43 (14.3%). In patients who did not receive antiseizure prophylaxis, 34 of 157 (21.6%) had an early seizure, whereas in patients who did receive antiseizure prophylaxis, 9 of 143 (6.3%) had an early seizure. Prophylactic antiseizure medication was associated with a reduced incidence of early seizures (adjusted odds ratio 0.25, 95% confidence interval 0.11–0.54, *P* = 0.0005) in patients at high risk for early seizures. There was no association between prophylactic medication use and modified Rankin Scale scores at three-month follow-up.

**Conclusions:**

In a retrospective analysis of a multicenter cohort of patients at elevated seizure risk after ICH, prophylactic antiseizure medication was associated with a reduced likelihood of an early seizure.

## Introduction

Acute intracerebral hemorrhage (ICH)—characterized by bleeding into brain tissue—frequently results in significant disability or death. Within the week following the onset of ICH, early seizures occur in approximately 10–25% of patients [1, 2]. In some cases, these early seizures may be correlated with additional complications, such as brain herniation, coma, and subsequent late seizures [3]. Seizures are associated with increased disability, mortality, and diminished health-related quality of life [3, 4]. Consequently, preventing early seizures emerges as a compelling strategy for improving outcomes following ICH [5]. However, it is not clear how to effectively prevent early seizures (e.g., within one week of ICH symptom onset).

Prophylactic antiseizure medications present a potential means to prevent early seizures. Previously, evidence-based guidelines recommended prophylactic phenytoin for patients with acute ICH [6]. However, subsequent findings suggested that phenytoin was associated with more side effects, fever, and worse outcomes compared with patients who did not receive prophylaxis [7, 8]. Levetiracetam has since become more commonly used and has fewer side effects than phenytoin [9]. Nevertheless, prophylactic levetiracetam is not without adverse effects and has been linked to lower cognitive function and health-related quality of life measures at follow-up when used indiscriminately [10]. More recent clinical guidelines suggest against the use of prophylactic antiseizure medication because of a lack of evidence of efficacy coupled with the adverse functional effects [2, 11]. These recommendations are based on studies involving patients with ICH and varying risk profiles for early seizure. Improved patient selection and targeted use of prophylactic antiseizure medication in selected patients with elevated risk of early seizure might be more likely to demonstrate efficacy for seizure prevention.

Preventing early seizures requires selecting an appropriate population of patients with elevated risk and assessing the efficacy of the prophylaxis within that population. Recent studies have advanced our ability to predict early and late seizures, allowing for precise identification of patients with ICH who may benefit from prophylactic antiseizure medications. For instance, the CAVE score predicts late seizures based on the factors of cortical (lobar) hematoma location (C), younger age (< 65 years) (A), higher hematoma volume (≥ 10 mL) (V), and early seizures (within one week of ICH) (E) [5]. Focusing on the C, A, and V subscores enhances predictions of early seizures. Among these risk factors, lobar hematoma most increases the likelihood of early seizure seizures after ICH and most drives physician decision-making regarding the decision to administer prophylactic antiseizure medication [12, 13]. Hence, we focus on lobar hematoma here.

Potentially efficacy could be estimated from clinical trials and observational studies. A recent prospective, randomized clinical trial (PEACH) investigated the efficacy of prophylactic levetiracetam, suggesting potential benefits in a highly selected patient population. However, the trial was ultimately halted early because of low recruitment, leaving it underpowered to estimate efficacy [14]. These recruitment challenges underscore the limitations of clinical trials in studying this group of highly selected patients, suggesting that observational studies may provide a better framework for exploring the association between antiseizure prophylactic medication and the development of early seizures.

In this study, we investigate this association through a retrospective analysis of a prospective observational study involving patients with spontaneous ICH upon hospital admission. We hypothesized that prophylactic antiseizure medications would be associated with a lower likelihood of early seizures in patients with an elevated risk for early seizures.

## Methods

### Patients

This is a retrospective analysis of a prospectively curated cohort. In the original prospective observational study, patients with spontaneous ICH were prospectively identified on hospital admission to investigate and predict hematoma expansion [15]. We prospectively recorded demographic information, medical history, standardized clinical assessments of severity (e.g., ICH Score, a composite of age, hematoma volume, location and level of consciousness [16]), and neuroimaging data. Seizures were diagnosed as previously reported, and the date was prospectively recorded [17]. We refer to “early seizures” as those that occur during the index hospital stay after admittance for spontaneous ICH (see Results Section). The occurrence of seizure medication as treatment or prophylactic was prospectively recorded.

### Identification of Patients with Elevated Risk

This study considered all patients with a lobar hematoma, the most frequently cited risk factor for early seizures [1, 5, 13, 18, 19].

### Data Collection

Participating sites were Northwestern Memorial Hospital (north side of Chicago, IL, A.M.N.), Northwestern Medicine Central DuPage Hospital (Winfield, IL, H.S.), University of Chicago Hospital (south side of Chicago, IL, F.G.), University of Iowa (Iowa City, IA, B.F.), and University of Texas at Houston (Houston, TX, T.R.C.). All sites used the same online case report forms for consistency. We prospectively recorded the dosing of any antiseizure medication as prophylactic or therapeutic. In this observational cohort, the administration of antiseizure medication was at the discretion of the treating physician and not a specific study protocol.

### Electroencephalogram Monitoring

Patients with ICH and reduced consciousness (e.g., not arousable to voice and following commands) underwent continuous electroencephalogram (EEG) monitoring by an electroencephalographer at the discretion of the treating physician. Consideration of discontinuing EEG monitoring was determined when further subclinical seizures were felt to be highly unlikely.

### Ethical Approval

The proposal was approved by the institutional review board (IRB). Patients provided consent for data acquisition. If the patient could not be consented because of neurologic injury, a legally authorized representative was asked to provide consent. If the patient died, the IRB approved enrollment with a waiver of consent.

### Outcomes Assessment

We obtained outcomes with the modified Rankin Scale (mRS) as previously described, using a validated questionnaire [20]. We used the Strengthening the Reporting of Observational Studies in Epidemiology reporting guideline for observational cohort studies [21].

### Statistical Analysis

Continuous data (e.g., age) are reported as median, whereas categorical data are expressed as percentages. Our primary aim in this retrospective analysis of the observational study data focused on lobar hematoma location as the risk factor used to identify the subset of patients with elevated risk of early seizure. We assessed the association between prophylaxis use and the binary outcome of early seizure incidence.

*χ*^2^ tests of independence were performed to evaluate the unadjusted association between categorical variables. Additionally, we conducted covariate-adjusted logistic regression models, with early seizure incidence as the binary outcome and antiseizure medication as the independent variable of interest, controlling for sex, age, and hematoma volume.

Covariate-adjusted ordinal regression models were conducted to examine the relationship between antiseizure medication and mRS scores (0–6) at three months after ICH. These models also controlled for sex, age, hematoma volume, and seizure status.

The statistical analysis was performed by M.B., using R v4/RStudio v1.4 and standard packages (base R, “dplyr”) [22].

## Results

### Elevated Risk Population Selection

Of the approximately 900 patients in the original prospective observational study, we selected the 300 study participants with lobar hematomas identified on neuroimaging at ICH symptom onset. The demographics and ICH scoring of the cohort are detailed in Table 1. Prophylactic antiseizure medications were administered to 143 patients, representing 48% of the patients with lobar hematoma (see Table 2). Notably, this proportion of patients receiving prophylaxis remained fairly stable throughout the data collection period of 2006–2023, despite the updated guidelines during this period.

**Table 1:** Subject characteristics stratified by Antiseizure Medication (ASM) administration status

	Did not receive ASM	Received ASM	Overall
	(*N* = 157)	(*N* = 143)	(*N* = 300)
*Sex*			
Female	75 (47.8%)	83 (58.0%)	158 (52.7%)
Male	82 (52.2%)	60 (42.0%)	142 (47.3%)
**Age (years)**	72.0 [62.0–80.0]	71.0 [61.0–79.5]	72.0 [62.0–80.0]
**ICH Volume (mL)**	24.0 [8.00–60.0]	32.0 [13.0–60.0]	27.0 [10.0–60.0]
*Data collection site*			
Northwestern Memorial Hospital	141 (89.8%)	131 (91.6%)	272 (90.7%)
Other	16 (10.2%)	12 (8.4%)	28 (9.3%)
*ICH score*			
0	43 (27.4%)	31 (21.7%)	74 (24.7%)
1	54 (34.4%)	28 (19.6%)	82 (27.3%)
2	15 (9.6%)	44 (30.8%)	59 (19.7%)
3	15 (9.6%)	30 (21.0%)	45 (15.0%)
4	20 (12.7%)	10 (7.0%)	30 (10.0%)
5	9 (5.7%)	0 (0%)	9 (3.0%)
6	1 (0.6%)	0 (0%)	1 (0.3%)
**EEG Performed**	63 (40.1%)	73 (51.0%)	136 (45.3%)
**History of ICH**	5 (3.2%)	9 (6.3%)	14 (4.7%)
**History of Ischemic Stroke**	22 (14.0%)	25 (17.5%)	47 (15.7%)
Drug use prior to admission*			
Aspirin	57 (36.3%)	48 (33.6%)	105 (35.0%)
Other antiplatelet	17 (10.8%)	16 (11.2%)	33 (11.0%)
Warfarin	14 (8.9%)	11 (7.7%)	25 (8.3%)
Direct oral anticoagulant	8 (5.1%)	4 (2.8%)	12 (4.0%)
Antiseizure medication (ASM)	1 (0.6%)	2 (1.4%)	3 (1.0%)
Cerebral amyloid angiopathy**	46 (29.3%)	42 (29.4%)	88 (29.3%)

n (%); Median [Q1–Q3]

^*^Subjects could be on multiple drugs prior to admission; percentages will not sum to 100%

^**^Clinical diagnosis suspected during admission by Modified Boston Criteria

**Table 2:** Patients with Lobar ICH, Stratified by Early Seizure and Prophylactic Antiseizure Medication

	Seizure		
Prophylactic Antiseizure Medication	No	Yes	Total
No	123	34	157
Yes	134	9	143
Total	257	43	300

Prophylactic antiseizure medication reduce the odds of experiencing an early seizure (chi-square test of independence *p*-value = 0.0003, aOR of 0.25 in covariate-adjusted logistic model with respective 95% CI 0.11–0.55 and *p*-value = 0.0006)

### Prophylactic Medication and Seizure Status

An early seizure occurred in 43 (14.3%) patients, with 2 patients (4.7%) exhibiting convulsive status epilepticus, and 11 (25.6) exhibiting nonconvulsive status epilepticus.The median number of days from ICH symptom onset to first seizure was one (interquartile range 0–2) day. All patients experienced their first seizure within a week of ICH symptom onset, with the exception of one patient who had a seizure on day 11 (Table 3).

**Table 3:** Patients with lobar ICH, Age < 65 and hematoma volume ≥ 10 mL, stratified by early seizure and prophylactic antiseizure medication

	Seizure		
Prophylactic antiseizure medication	No	Yes	Total
No	24	30	54
Yes	7	2	9
Total	31	32	63

Analysis suggestive of association between prophylactic medication and reduced occurrence of early seizures (chi-square test of independence *p*-value = 0.13, aOR of 0.208, CI: 0.037–1.164, in covariate-adjusted logistic model with respective *p*-value = 0.07)

**Fig. 1:**
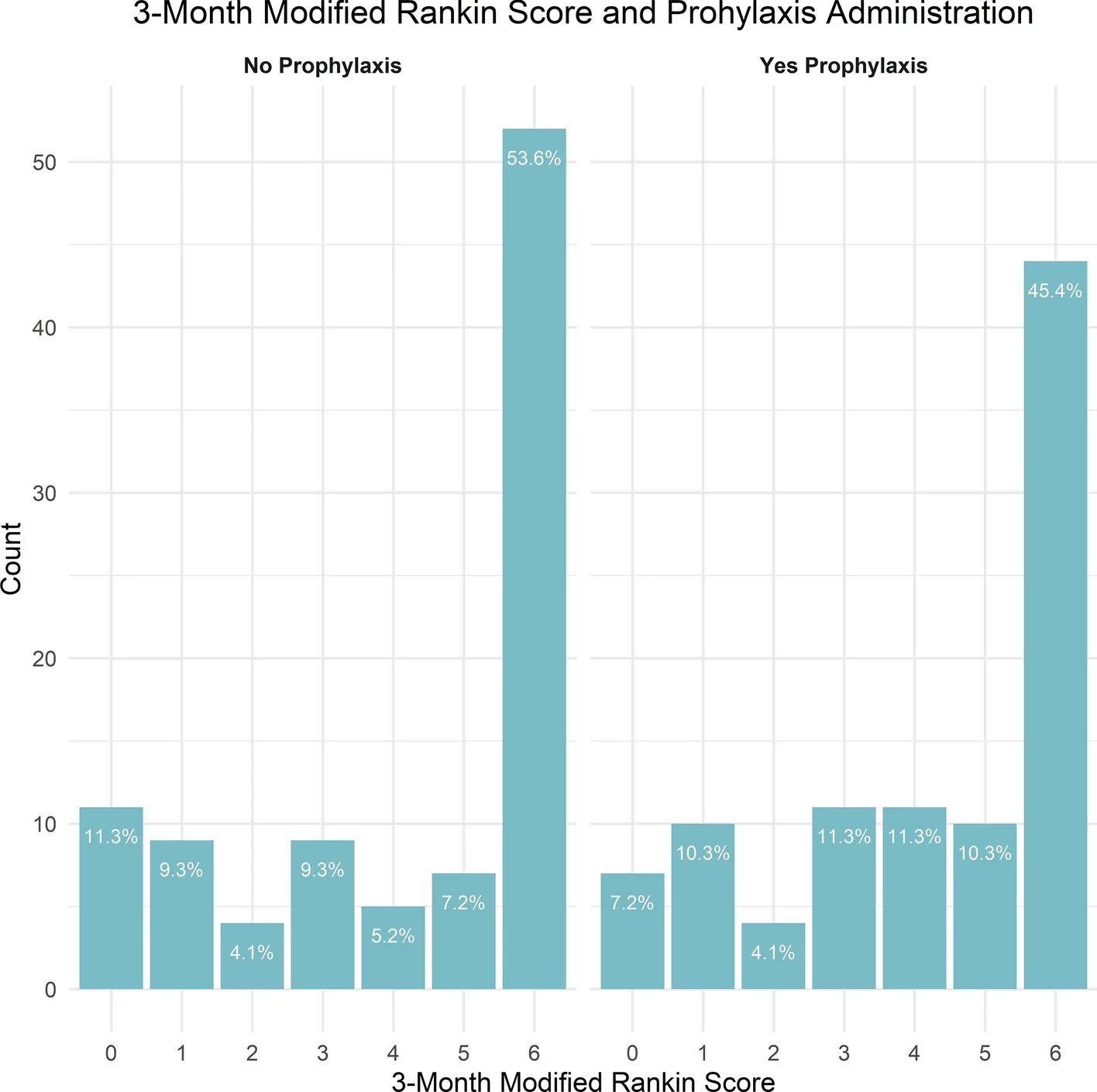
Distrbution of modified Rankin Scale scores for patients with and without antiseizure prophylactic medication, collected at 3-month follow-up after intracerebral hemorrhage

In covariate-adjusted logistic regression models, the use of prophylactic antiseizure medications was statistically significantly associated with a reduced risk of seizures after controlling for sex as a biological variable, and binary indicators of younger age (age < 60 years) and large hematoma volume (volume ≥ 10 mL) (adjusted odds ratio 0.250, confidence interval 0.11–0.55, *P* = 0.0006). In this model, neither younger age (*P* = 0.10) nor large hematoma volume (*P* = 0.11) was significantly associated with the occurrence of early seizure. Notably, mRS scores did not show statistically significant differences between the prophylaxis medication groups at three-month follow-up, even after adjusting for seizure status (covariate-adjusted *P* value = 0.64) (Fig. 1).

### Prophylactic Medication and EEG Monitoring

Among the 300 patients with lobar hematomas, 136 patients (45.3%) underwent continuous EEG monitoring. Of those monitored, 73 patients (53.6%) received prophylactic antiseizure medication. *χ*^2^ tests of independence indicated no statistically significant association between prophylaxis medication and monitoring status (*P* = 0.07). Additionally, there was no statistically significant association between prophylaxis use and nonconvulsive seizure detected on EEG monitoring: Among the 11 patients with nonconvulsive status epilepticus, 5 (45.5%) received prophylactic antiseizure medication.

There were 17 patients with a documented history of seizures in the cohort, and 3 patients with documented history of receiving prophylaxis. Results were similar regardless of the inclusion or exclusion of these patients.

## Discussion and Conclusion

In a retrospective analysis of a prospectively curated cohort of patients at elevated risk for early seizures after ICH (i.e., patients with lobar hematomas), we found that prophylactic antiseizure medication was associated with fewer early seizures. The results from this observational study suggest that future research to prevent seizures might evaluate prophylactic antiseizure medication on patients at elevated risk, specifically by virtue of hematoma location as a convenient entry criterion.

Although lobar hematoma location is useful to predict a higher risk of early seizures, it is still not exact. All the patients in this cohort had a lobar hematoma location, yet early seizures only occurred in about one in five patients without antiseizure prophylaxis. Adding the additional known risk factors of age and hematoma volume did not substantially change our results. Additional methods to precisely predict seizures and select patients for prophylactic antiseizure medications are needed.

The optimal strategy for the use of prophylactic antiseizure medication remains controversial. We have previously noted that there is wide variability the use of antiseizure medication [23], which is in line with this uncertainty. Future protocols will need to minimize variation to estimate potential efficacy.

In this article, we counted seizures that were detected clinically (e.g., a typical clinical seizure observed in the neurological intensive care unit) and those detected on EEG. This cohort had a relatively low rate of subclinical seizures detected on EEG alone. It is not clear whether the potential benefits of prophylactic antiseizure medication are restricted to clinical or subclinical seizures. Additionally, because nearly all seizures were within seven days in this study, with a median time of two days, a short time of antiseizure prophylaxis might be reasonable. However, effects on the incidence of late (> 7 days) are not known at this time.

There are limitations to this work. The administration of prophylactic antiseizure medication was unblinded and at the discretion of the treating physician. However, there is no evidence that clinicians are better at predicting seizures than the risk factors for which we accounted. In adaptive decision choice experiments, clinicians chose to administer prophylactic antiseizure medications primarily based upon lobar hematoma location [12], one reason we chose that as the screening criterion. The mRS did not vary with prophylactic antiseizure medication. Although the use of antiseizure medications can lead to a decline in quality of life due to side effects such as fatigue, cognitive impairment, and mood changes, the mRS is generally insensitive to these domains [24]. We focused our attention on early seizures, given the impact of early seizures on hospital-related outcome. Future studies will need to assess whether the prevention of early seizures reduces the risk of late seizures and epilepsy after the acute hospitalization. Late seizures are more challenging to assess because they require patient assessment and accurate reporting, and patient self-report is often inaccurate [25].

Strengths of this work include the use of multicenter data, which suggests these data are generalizable across several metropolitan areas. We also prospectively identified patients, used the same case report forms for harmonization, and prospectively assessed valid outcomes. The results are consistent with prior reports that suggest prophylactic antiseizure medications are likely to prevent seizures in patients at high risk [14].

In sum, we found that prophylactic antiseizure medication was associated with a reduced risk of early seizures in patients with a lobar hematoma location. Future research might focus on patients at elevated risk for seizures from lobar hematoma to determine the efficacy of prophylactic antiseizure medication in patients at elevated risk.
